# Learning Curve and Detection Accuracy of Sentinel Lymph Node Biopsy for Endometrial Cancer Staging: Experience from an ESGO & GESEA Certified Center

**DOI:** 10.3390/medsci14060617

**Published:** 2026-09-30

**Authors:** Grigoris Grimbizis, Dimitrios Zouzoulas, Theodoros Theodoridis, Ioanna Vagioni, Panagiotis Tzitzis, Kimon Chatzistamatiou, Dimitrios Tsolakidis

**Affiliations:** 1st Department of Obstetrics & Gynecology, Aristotle University of Thessaloniki, “Papageorgiou” Hospital, 56403 Thessaloniki, Greece

**Keywords:** endometrial cancer, sentinel lymph node biopsy, learning curve, detection rate

## Abstract

**Background/Objectives**: Pelvic lymphadenectomy has long been considered the gold standard for surgical staging in early-stage endometrial cancer. However, sentinel lymph node biopsy (SLNB) with ultrastaging has emerged as a less invasive alternative. The aim of the study is to assess the detection rate, learning curve and diagnostic accuracy of SLNB in a cohort of patients with a high prevalence of obesity and early-stage endometrial cancer. **Methods**: This relatively small prospective single-center study included patients with presumed early-stage endometrial carcinoma who underwent laparoscopic hysterectomy, SLNB and bilateral pelvic lymphadenectomy at the 1st Department of Obstetrics & Gynecology between 1 April 2023 and 31 May 2025. **Results**: Forty-one patients were recruited. The mean age was 64.3 years, and the mean BMI was 32.4 kg/m^2^. Bilateral and overall sentinel lymph node (SLN) detection was achieved in 27 of 41 patients (65.9%) and 85.4%, respectively. Concerning the learning curve, after the first 20 cases the bilateral detection rate was as high as 90.5% (*p*-value < 0.001). The median number of SLN for the left and right side were 1 (IQR: 1–2) and 2 (IQR: 1–3), respectively. The median number of lymph nodes dissected by lymphadenectomy was 7 (IQR: 5–8) for both sides. There were three patients with lymph node metastases, all of which were detected by SLNB. **Conclusions**: Bilateral detection of SLNs is technically feasible and requires a relatively short learning curve, under expert supervision in a high-volume, standardized training environment, even in patients with a high prevalence of obesity. The adoption of a standardized detection protocol is key to avoiding mapping failure.

## 1. Introduction

Endometrial cancer (EC) ranks among the most frequently diagnosed malignancies in women and is a notable contributor to cancer-related mortality among women worldwide [1]. Age-standardized incidence rates vary widely across regions, ranging from approximately 1 to 30 new EC cases per 100,000 women [2]. Contemporary epidemiologic series consistently demonstrate a progressive increase in incidence across age groups [3]. The rising prevalence of obesity remains the predominant factor driving this increase in EC incidence [4], while additional risk factors reflect prolonged exposure to unopposed estrogen, including early menarche, late menopause, tamoxifen use, nulliparity, infertility, and polycystic ovary syndrome [5]. Because symptoms often occur early in the disease course, most women with EC are diagnosed at an early stage. Postmenopausal bleeding is the predominant presenting symptom, whereas premenopausal women more commonly present with intermenstrual bleeding or abnormally heavy menstrual bleeding [6].

Early-stage EC accounts for approximately 67% of cases and is associated with an 81% 5-year overall survival (OS), whereas 5-year OS declines to 17% and 15% for stage IVa and IVb disease, respectively [7]. Nodal metastasis is associated with poor prognosis and typically necessitates adjuvant therapy [8]. Importantly, pelvic lymphadenectomy, independently of adjuvant therapy, does not confer a survival benefit in EC [9]. Consequently, sentinel lymph node biopsy (SLNB)—the removal of the first lymph node to receive lymphatic drainage from the tumor site—has been proposed as a less invasive strategy for nodal assessment [10].

Sentinel lymph node (SLN) mapping, most commonly performed using indocyanine green (ICG) and fluorescence imaging, has become well established as an alternative to complete pelvic lymphadenectomy for staging early-stage EC [11]. In the era of SLNB for EC, it is essential that surgeons adopt a standardized SLN detection protocol and follow a defined learning curve, during which pelvic lymphadenectomy is performed. Several cut-off points have been proposed for the SLNB learning curve in EC [12,13] to achieve an acceptable bilateral mapping rate (≥75%) [14]. Furthermore, SLN mapping may be influenced by patient-related factors, particularly obesity, which is highly prevalent among women with EC. Increasing body mass index (BMI) has been associated in several studies with lower bilateral mapping rates, higher mapping failure rates, and a greater likelihood of empty-packet dissection [15]. These findings suggest that excess adiposity may impair tracer migration and intraoperative visualization of sentinel nodes, indicating that higher BMI may negatively affect technical success and warrant specific preoperative counseling and operative planning [16].

The primary objective of this study was to evaluate the detection rate and learning curve of SLNB in the pelvic area and to assess its diagnostic accuracy for identifying lymph node metastasis during laparoscopic staging with ICG mapping in a cohort of patients with a high prevalence of obesity and presumed early-stage EC.

## 2. Materials and Methods

This prospective cohort study was conducted in the Gynecological–Oncology Unit of the 1st Department of Obstetrics & Gynecology, Aristotle University of Thessaloniki, “Papageorgiou” General Hospital, from 1 April 2023 to 31 May 2025. The department has long-standing experience in minimally invasive gynecologic oncology and serves as a European Society of Gynaecological Oncology (ESGO) Accredited Center in Gynecological Oncology Training and as an Accredited Center for the Gynaecological Endoscopic Surgical Education and Assessment (GESEA) Expert Level Oncology Diploma. All procedures were performed by a single fellow under the supervision of three senior gynecologic oncologists experienced in endoscopic surgery. Specifically, the fellow had no prior experience in SLNB but was required to have obtained the GESEA level 1 and level 2 certificates to ensure adequate competence in laparoscopic surgery. In contrast, the three senior surgeon-mentors each had experience of more than 100 SLN detection procedures.

**Figure 1:**
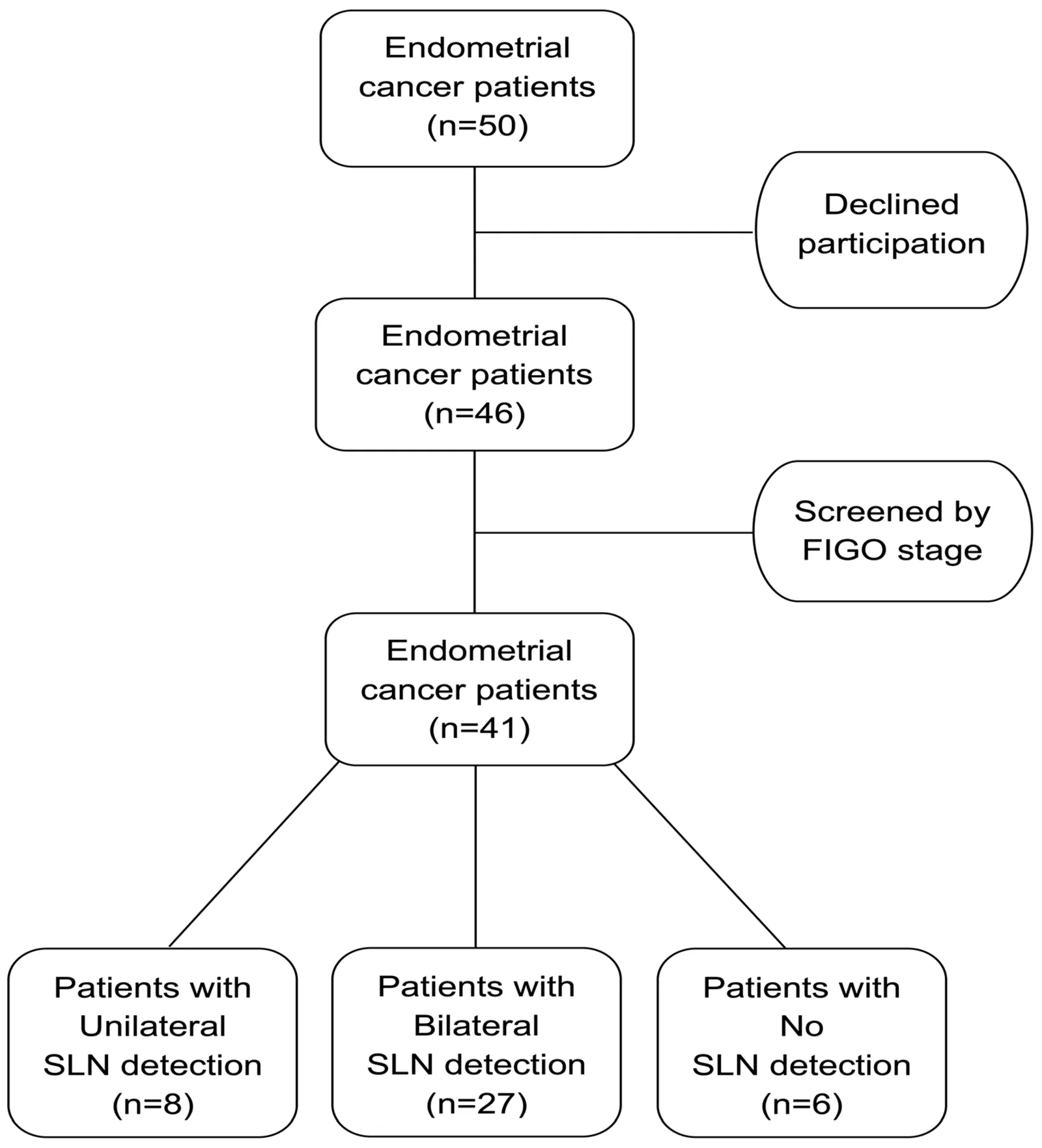
Patient study flowchart.

Patients with presumed early-stage low-risk endometrial cancer, corresponding to International Federation of Obstetrics and Gynecology (FIGO) stages I–II were enrolled [17]. Of the 50 patients initially recruited, five were excluded because preoperative imaging revealed a higher FIGO stage, and four were withdrawn because they revoked consent and declined participation immediately before surgery. Preoperative evaluation included pelvic magnetic resonance imaging (MRI) to assess myometrial invasion, cervical stromal involvement and locally advanced disease, as well as computed tomography (CT) of the thorax and abdomen for staging purposes. Histopathological diagnosis and tumor grading were established preoperatively from endometrial biopsy specimens obtained via hysteroscopy or dilatation and curettage (D&C). All patients underwent laparoscopic total hysterectomy with bilateral salpingo-oophorectomy and pelvic SLNB, followed by bilateral pelvic lymphadenectomy. Obesity was not an explicit inclusion criterion. The patient flowchart is presented in Figure 1.

SLNB was performed according to a standardized, anatomically guided protocol [18,19]. A solution of ICG at a concentration of 2.5 mg/mL in sterile water was injected into the uterine cervix at two sites (3 and 9 o’clock) using a 22G spinal needle (Spinocan^®^, B. Braun Melsungen AG, Melsungen, Germany), with 1 mL injected superficially and 1 mL deeply on each side (for a total volume of 4 mL). Cervical injection was followed by the creation of pneumoperitoneum, primary access to the abdominal cavity, placement of secondary trocars, and exploration of the pelvis, allowing adequate time for ICG mapping for up 30 min after injection. The IMAGE1 S 4U RUBINA endoscopic imaging platform (KARL STORZ SE & Co. KG, Tuttlingen, Germany) was used. Two of the three OPAL1 NIR/ICG visualization modes (Intensity Map and Monochromatic) were primarily employed to visualize the fluorescence signal along the lateral pelvic peritoneum. If no ICG-positive lymphatic channel was observed, particularly in obese patients, a cervical reinjection of 1 mL of ICG solution was performed on the unmapped side.

Regardless of initial visualization, the retroperitoneum was opened from the border of the round ligament, followed by the systematic development of the retroperitoneal spaces and identification of anatomical landmarks. SLNs were then identified based on the fluorescence signal as the lymph nodes closest to the uterus demonstrating ICG uptake with an associated ICG-positive afferent lymphatic channel. Troubleshooting in cases of absent SLN identification included observation over time, contralateral exploration, and, if necessary, more extensive retroperitoneal dissection. If no SLN was detected after completion of troubleshooting, or after recognition and excision of the sentinel nodes, pelvic lymphadenectomy was performed as a backup procedure in accordance with the study protocol.

Histological assessment included ultrastaging of SLNs using hematoxylin and eosin (H&E) staining and cytokeratin AE1/AE3 immunohistochemistry [20]. After routine H&E evaluation of each SLN, any node negative on initial review was sectioned to obtain two additional 5-µm sections at each of two deeper levels, 50 µm apart. At each level, one slide was stained with H&E and one with cytokeratin AE1/AE3 immunohistochemistry, yielding a total of five slides per block. Patients with isolated tumor cells (ITCs) were considered node positive. This decision was based on the results of a recent multicenter retrospective study [21], in which low-risk patients with ITCs who did not receive adjuvant therapy had worse recurrence-free survival and an increased risk of non-vaginal recurrence. Written informed consent was obtained from all patients before surgery and the study protocol was approved by the Institutional Scientific Committee (No. 2023-B2015-246/approved date: 10 March 2023).

Statistical analyses were performed using R software, version 4.3.0. The study was designed as an exploratory feasibility and learning-curve study rather than a confirmatory trial; therefore, no formal power calculation was undertaken. The sample size included all consecutive eligible patients treated by a single fellow at this center during the predefined implementation phase of SLNB. Based on previously published literature and the longest learning-curve plateau, the target sample size was set at approximately 40 cases. Extending pelvic lymphadenectomy beyond the implementation phase solely to increase the sample size was judged to expose patients to unnecessary morbidity and was therefore ethically unacceptable. Categorical variables were summarized using descriptive statistics and presented as absolute frequencies and percentages, whereas continuous variables were summarized using measures of central tendency (mean, median) and dispersion (range, standard deviation). Differences in SLN detection rates between the two study periods were assessed using Pearson’s chi-square test. Logistic regression and LC-CUSUM analyses were performed to further evaluate surgical competency in SLNB. No missing values were present in the dataset because all parameters were extracted from an online registry system. A two-sided *p*-value < 0.05 was considered statistically significant.

## 3. Results

A total of 41 patients with presumed early-stage EC were included in the study. Baseline characteristics are presented in Table 1. The mean age and body mass index (BMI) were 64.3 years and 32.4 kg/m^2^, respectively, indicating that most women in this cohort were obese. However, when BMI was analyzed as a continuous variable in relation to bilateral SLN detection, the results showed a trend toward lower detection rates as BMI increased, but this association was not statistically significant (OR: 1.02, 95% CI: 0.90–1.16, *p* = 0.748. Most patients (approximately 80%) had a good performance status (PS: 0–1) and an acceptable American Society of Anesthesiologists Physical Status Classification System (ASA score: 1–2), reflecting thorough preoperative evaluation and optimization of comorbidities. Based on preoperative histological reports, nearly all patients had low-grade endometrioid EC. Central pathological review of both the initial endometrial biopsy and the final uterine specimen revealed no discrepancies in tumor grade or histological subtype.

Analyzing the postoperative characteristics of the enrolled patients, the median surgical duration was 180 min (IQR: 150–200) and the median intraoperative blood loss was 100 mL (IQR: 100–200). Unilateral and bilateral SLN detection were achieved in 8 of 41 (19.5%) and in 27 of 41 patients (65.9%), respectively. SLN detection in at least one hemipelvis (meaning the overall detection rate, calculated as the sum of unilateral and bilateral detection rates) was observed in 35 of 41 patients (85.4%) of the population. The median number of SLN retrieved on the left side was 1 (IQR: 1–2) and on the right 2 (IQR: 1–3). The median number of dissected lymph nodes during pelvic lymphadenectomy was 7 (IQR: 5–8) for both pelvic sides. In the majority of cases (75.6%) lymph node specimens were collected using an endobag. According to the 2023 FIGO classification, only 73.2% of patients had stage I disease on final pathology. This was because three patients were upstaged to stage III disease after SLNB and pelvic lymphadenectomy, because of lymph node metastases. These data are summarized in Table 2.

**Figure 2:**
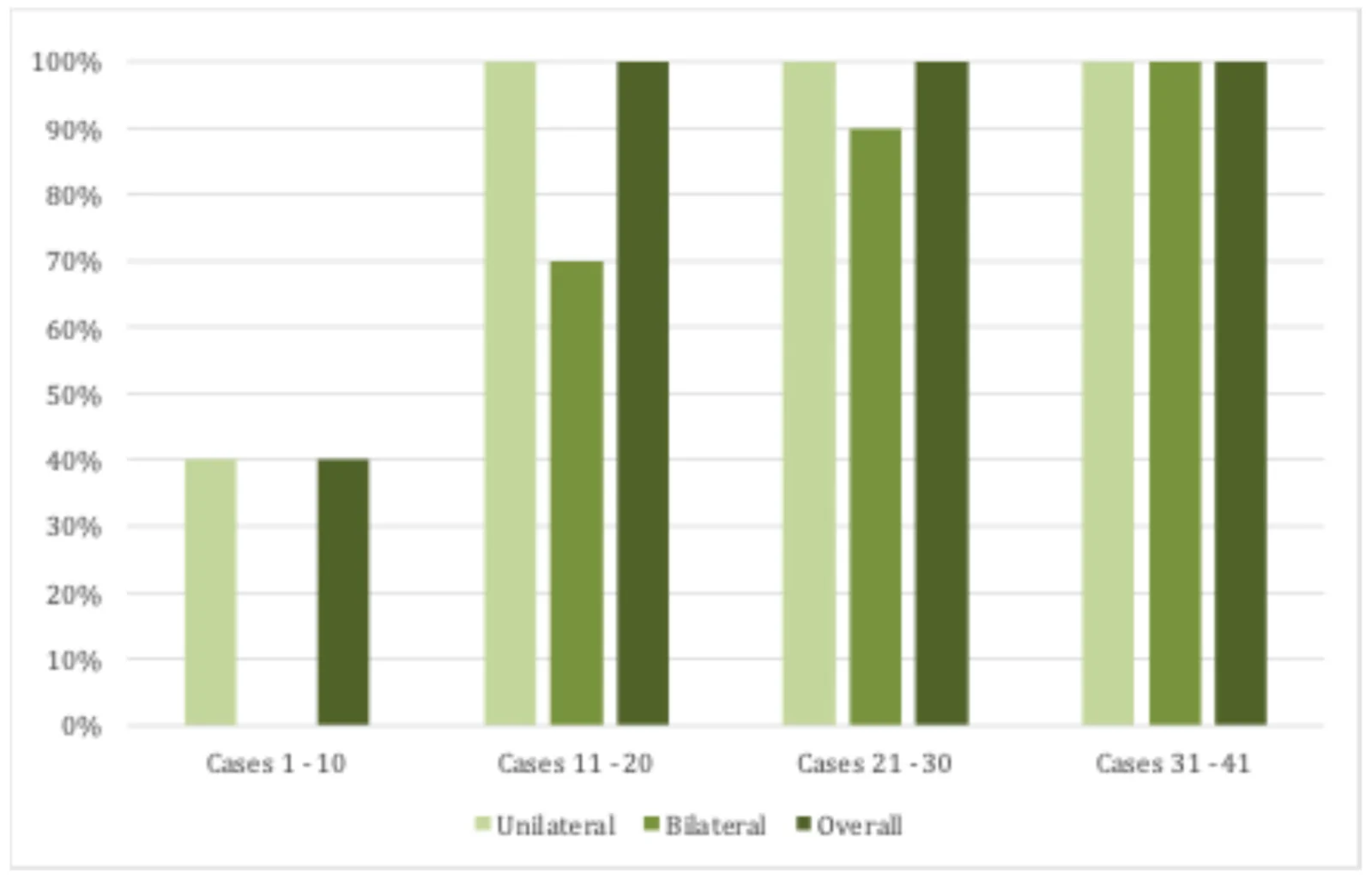
Chronological SLN detection rate.

**Figure 3:**
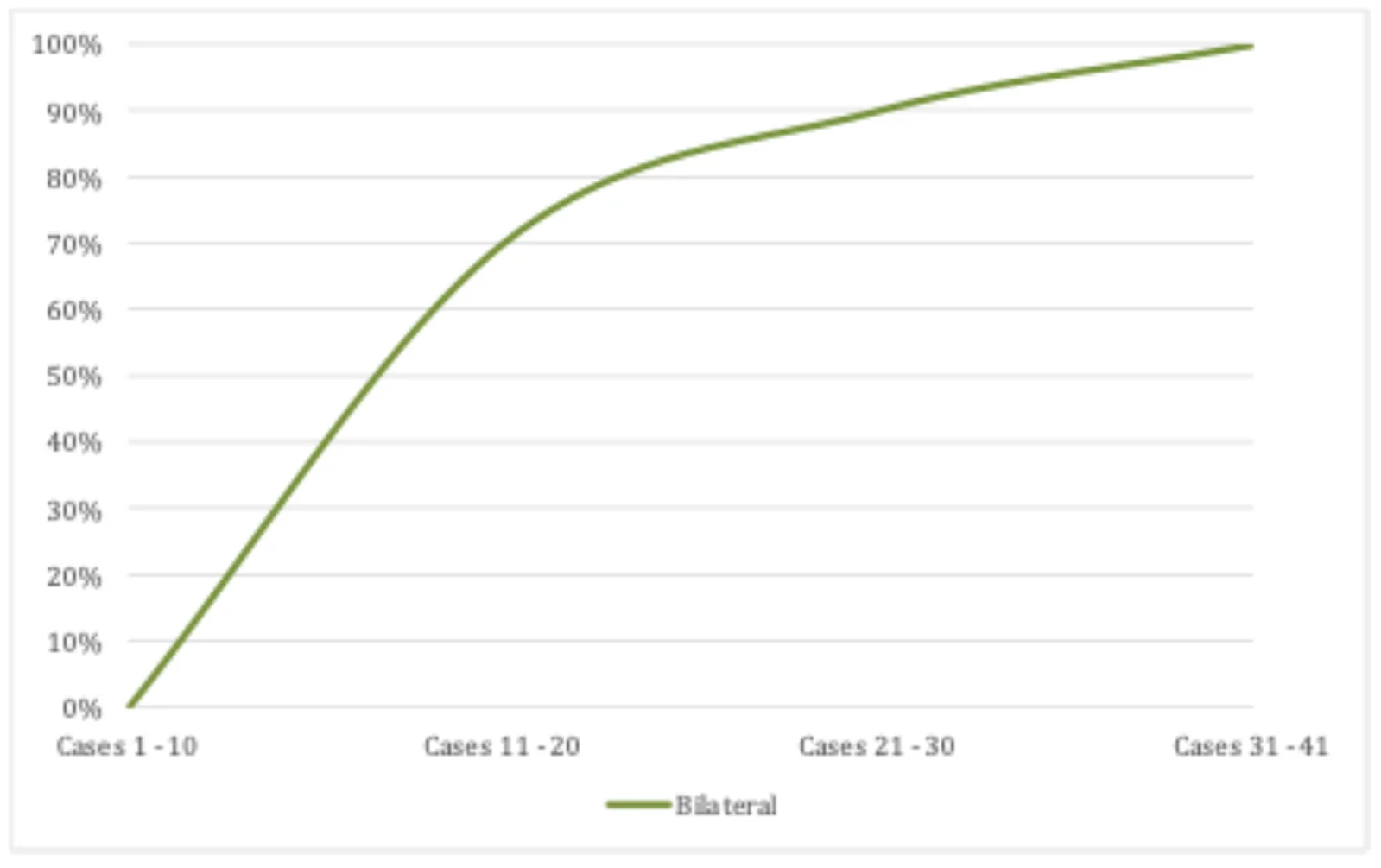
Learning curve for bilateral SLNB.

Regarding the learning curve for SLNB training, the study cohort was divided chronologically into four sequential groups, each consisting of ten consecutive patients (with the last group including eleven patients to capture all forty-one cases). Unilateral, bilateral and overall detection rates were calculated for each group and these data are presented in Figure 2. In the first group, overall and bilateral SLN detection rates were low, at 40% and 0%, respectively. Notably, six patients had complete mapping failure and as specified in the protocol all underwent pelvic lymphadenectomy, which did not reveal any positive lymph nodes on final histopathological examination. Among the subsequent 21 patients (cases 21–41), bilateral detection rate increased to 70% (cases 21–30) and reached 90% (cases 31–41). To further evaluate the impact of increasing SLN experience on detection rates, the cohort was also divided into two periods. Among the first 20 patients, an SLN was identified bilaterally in 8/20 patients (detection rate: 40%), whereas among the subsequent 21 patients (cases 21–41), the bilateral detection rate increased significantly to 19/21 patients (90.5%) (*p*-value < 0.001). The learning curve for bilateral SLN detection is illustrated in Figure 3.

**Figure 4:**
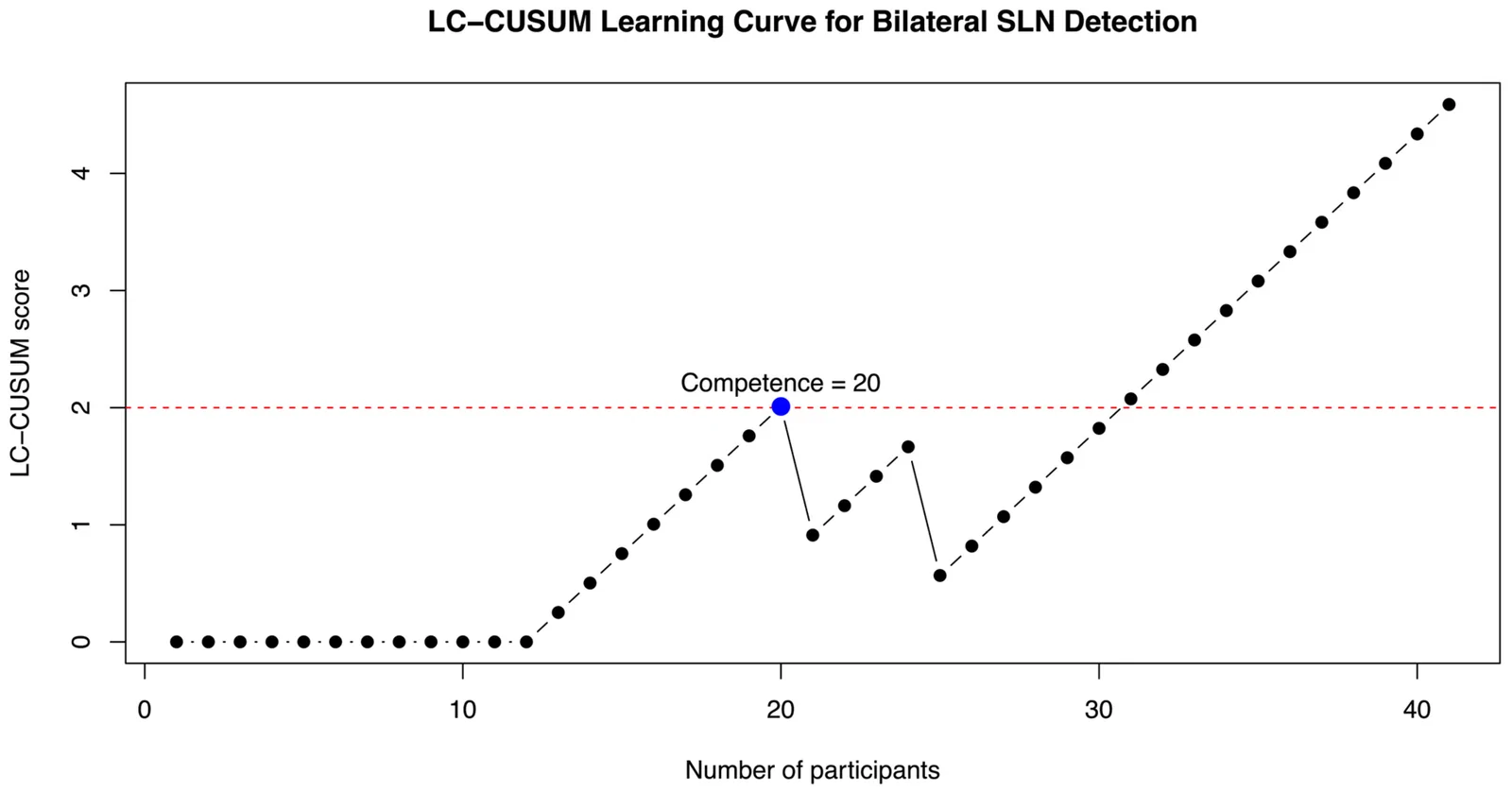
LC-CUSUM learning curve for bilateral SLNB.

Moreover, we conducted a logistic regression analysis using case order as a surrogate for the learning curve to assess its association with bilateral SLN detection. The results indicated that for every additional SLNB case performed by the surgeon, the odds of successful bilateral SLN detection increased by approximately 33% (OR: 1.33, 95% CI: 1.11–1.59, *p* = 0.002). Finally, the LC-CUSUM learning curve for bilateral SLN detection was used to assess the progression of surgical performance during sequential procedures. An unacceptable failure rate of p_0_ = 0.30 and an acceptable failure rate of p_1_ = 0.10 were predefined. The LC-CUSUM threshold was defined according to these parameters and error levels (α = 0.05 and β = 0.10), resulting in h = 2. The LC-CUSUM score remained at the zero boundary during the first 12 cases, because of consecutive failures. From case 13 onward, the score increased progressively, reflecting improved surgical performance. Two isolated failures occurred in cases 21 and 25, but these did not alter the overall upward trend. The curve reached the predefined threshold after 20 surgical procedures, suggesting that the surgeon achieved a stable and reproducible technical performance in SLNB. These results are presented in Figure 4.

Additionally, a total of 73 SLNs were dissected from the right hemipelvis and 65 from the left. Most SLNs were identified near the external iliac vessels or superior to the obturator nerve. Detailed anatomical locations of all SLNs are provided in Table 3. In total, five positive pelvic lymph nodes were identified in three patients: two patients had bilateral pelvic lymph node metastases and one had unilateral involvement. With respect to the type of lymph node involvement, ultrastaging revealed bilateral micrometastases in one patient, whereas ITCs were present unilaterally in one patient. All patients underwent pelvic lymphadenectomy after SLNB, but no additional positive lymph nodes were detected. These findings are detailed in Table 4.

Moreover, all positive lymph nodes were identified through SLNB and ultrastaging resulting in 100% sensitivity (95% CI: 29–100), 100% specificity (95% CI: 91–100), 100% of positive predictive value (PPV) (95% CI: 29–100), 100% of negative predictive value (NPV) (95% CI: 91–100) and a 0% of false negative rate (FNR) (95% CI: 0–71).

## 4. Discussion

This prospective cohort study evaluated the detection rate and learning curve of SLNB in patients with presumed early-stage endometrial cancer and assessed the diagnostic accuracy of SLNB for pelvic nodal metastasis. By comparing SLNB with standard pelvic lymphadenectomy, the study had two main aims: first, to identify the optimal cut-off point in the SLNB learning curve in expert centers, and second, to assess the role of SLNB compared with lymphadenectomy in a cohort of patients with a high prevalence of obesity, with the aim of providing equivalent diagnostic accuracy while reducing morbidity and optimizing surgical staging strategies.

In our cohort forty-one patients were included. Most had mild to moderate comorbidities, according to their PS and ASA scores, with a mean age of 64 years and a mean BMI of 32 kg/m^2^. Although obesity was not an inclusion criterion, the results indicate that the majority (approximately 75%) of patients were obese, including four patients with a BMI greater than 40 kg/m^2^. Existing literature indicates that obesity does not negatively affect SLNB [22,23] but increases the risk of mapping failure [24]. Nevertheless, in our cohort the bilateral and overall (sum of unilateral and bilateral) SLN detection rates were 65.9% and 85.4%, respectively and logistic regression did not demonstrate a statistically significant association between BMI and bilateral SLN detection. Notably, although our study included patients with higher BMIs than many previous studies in the literature, the acceptable detection rates highlight that obesity may impede SLNB, but is not an insurmountable obstacle. In this cohort, increasing BMI was not statistically associated with bilateral SLN detection; however, the study was not powered to establish the independent effect of BMI on mapping success. Although higher BMI may make SLN mapping more technically challenging, these obstacles may be overcome through use of a standardized ICG-based SLNB mapping protocol. Moreover, it is important to state that the three patients that were upstaged to stage III disease on final histopathological examination, had initially been considered to have stage I disease on preoperative imaging; otherwise, they would not have been excluded from the study.

In the context of the current literature, our outcomes are comparable to those of the four landmark studies [25,26,27,28], that established SLNB for lymph node staging in endometrial cancer. However, because of the low prevalence of positive SLNs in our study, the results should be interpreted with caution with regard to the feasibility of implementing SLNB in the treatment algorithm, because our findings primarily add to evidence already established by large, well-powered studies. The French multicenter SENTI-ENDO trial [25], which included 125 participants, reported bilateral and overall SLN detection rate similar to those observed in our study, at 61.6% and 88.8%, respectively. However, the study population was only mildly overweight, with a mean BMI of 27 kg/m^2^, representing a significant difference compared with our study, in which obesity may have made SLN detection much more challenging. Furthermore, the FIRES trial [26], a US multicenter study of 340 patients, reported a lower bilateral SLN detection rate of 52%, but with a comparable overall rate of 86%; however, BMI characteristics of the study population were not reported. to our findings. Interestingly, the SHREC trial [27], a Swedish multicenter study including 257 cases, reported bilateral and overall SLN detection rate of 93.8% and 98.8%, respectively. These results may be explained by two main differences compared with our study: first, all patients underwent robotic surgery; and second the study did not report the mean BMI, although BMI > 40 kg/m^2^ was an exclusion criterion. Similarly, the SENTOR trial [28], the most recent Canadian multicenter study with 156 patients, also reported high SLN detection rates, with a bilateral and overall rates of 77.6% and 97.4%, respectively. Possible key factors underlying these high rates, compared with our findings, include the extensive experience from the senior surgeons who participated in the study and the lower median BMI of the population (27 kg/m^2^).

With regard to the learning curve for bilateral SLN detection in endometrial cancer, the main scientific focus of this study, our data demonstrate that, after the first 20 cases, the 75% cut-off point, suggested by ESGO surgical quality indicators [14], could be achieved. Specifically, although the bilateral detection rate in the whole cohort was 65.9%, this rate increased significantly to 90.5% among cases 21–41. Furthermore, the rate of bilateral successful SLN mapping significantly increased from 40% to 90.5% (*p*-value < 0.001), between cases 1–20 and cases 21–41. Most importantly, the LC-CUSUM analysis demonstrated that SLNB competency could be reached at approximately case 20. Previous studies investigating the learning curve of SLN mapping concluded that 30 cases were needed to reach an acceptable bilateral detection rate [12,29,30], while others extended the cut-off point to 40 cases [13,31], to safely omit pelvic lymphadenectomy. Our findings, which suggest a shorter learning curve (20 cases), than that reported in the existing literature, could be explained by two factors. First, the use of a standardized SLNB protocol with predefined actions for troubleshooting (re-injection, etc.), in the event of SLN mapping failure. Second, training in an expert setting, an ESGO and GESEA certified center, under the supervision of three senior surgeons experienced in SLNB.

Moreover, all three node-positive patients were identified by SLNB in this exploratory cohort; however, the very small number of metastatic cases precludes reliable estimation of diagnostic accuracy. Specifically, one patient had unilateral nodal metastasis and two had bilateral nodal involvement, resulting in 100% sensitivity, 100% specificity, 100% NPV, 100% PPV and a 0% FNR. These findings are consistent with those of the afore-mentioned cornerstone studies (SENTI-ENDO, FIRES, SHREC, SENTOR) [25,26,27,28], which reported similar high sensitivity and NPV rates, leading to the conclusion that SLNB, even among obese patients, may be a sensitive and accurate method for nodal assessment in early-stage disease, with high bilateral and overall detection rates. However, when SLN mapping failure occurs, a side-specific pelvic lymphadenectomy should be performed.

In addition, our findings further support the use of ultrastaging for SLNs, which allows the detection of microscopic metastatic deposits (micrometastases and ITCs) that could be overlooked by conventional pathology. One patient with bilateral nodal involvement had ITCs and was considered node positive. This is consistent with previous data, which suggest that SLNs may harbor low-volume metastases, which would not be detected by traditional histopathological examination of the lymph nodes [20]. The prognostic relevance of these findings remains under investigation, although early evidence suggests an association with an increased relative risk of recurrence, even following adjuvant therapy [32]. However, this should be interpreted with caution, because in the era of molecular classification, emerging analyses suggest that the prognostic significance of ITCs is modulated by tumor subtype, supporting a more nuanced, individualized approach to the interpretation of SLNB findings.

This study is particularly strengthened by its prospective design and standardized SLNB protocol, with clear troubleshooting steps in the event of mapping failure. The use of an online medical records system minimized missing data and improved data reproducibility. Nevertheless, several limitations must be acknowledged. First, this was a non-randomized trial with possible selection bias and no long-term oncologic outcomes or survival data. The principal limitation is the small sample size, which resulted in only three patients with lymph node metastases. This may affect the generalizability and statistical power of the study for both diagnostic accuracy and learning-curve analyses. However, once SLN detection rates in the last 21 cases exceeded 90%, it was considered ethically unacceptable to continue performing pelvic lymphadenectomy solely for training purposes. Another important consideration is that SLNB training, which was at an ESGO & GESEA certified center. This setting offers an excellent opportunity for a fellow to acquire SLNB skills within approximately 20 cases, but similar learning curves may not be achievable in routine practice at lower-volume centers. This observation underscores the need for dedicated fellowship training in gynecologic oncology within expert, certified centers. Finally, para-aortic lymphadenectomy was not routinely performed, limiting the assessment of SLN accuracy in the para-aortic region.

This study adds to the existing literature supporting SLNB as a safe and effective tool for nodal assessment in early-stage endometrial cancer, particularly among obese patients, with a relatively short learning curve in expert training centers. Future studies should focus on clarifying the importance of SLNB in the era of molecular classification in endometrial carcinoma. Further prospective multicenter studies evaluating the learning curve of SLNB are needed to validate the findings from this single-expert center study.

## 5. Conclusions

Based on the existing literature and the observed detection rates and diagnostic accuracy for pelvic nodal disease, SLNB could be considered as an alternative to pelvic lymphadenectomy for surgical staging in a cohort of patients with a high prevalence of obesity and presumed early-stage endometrial cancer. However, all ESGO quality indicators should be met, before initiating an SLN mapping program. Under expert supervision in a high-volume, standardized training environment, SLNB appears to require a relatively short learning curve. A standardized surgical protocol should be adopted, particularly one incorporating predefined troubleshooting measures in the event of mapping failure. Nevertheless, given the study’s small sample size, which was limited for ethical reasons, these results should be interpreted with caution and the adoption of SLNB should be individualized at the center and surgeon levels.

## Figures and Tables

**Table 1:** Baseline characteristics.

		Number of Patients (N)	Percentage (%)
Age (years)		mean: 64.3	SD: 9.9
BMI (kg/m^2^)		mean: 32.4	SD: 5.6
	25–30	10	24.4
	30–35	17	41.5
	35–40	10	24.4
	>40	4	9.7
Performance status			
	0	15	36.6
	1	20	48.8
	2	4	9.7
	3	2	4.9
ASA score			
	1	13	31.7
	2	21	51.2
	3	7	17.1
Histological types			
	endometrioid	41	100
	other	0	0
Tumor grade			
	low	35	85.4
	high	6	14.6

**Table 2:** Postoperative characteristics.

		Number of Patients (N)	Percentage (%)
Surgery duration (min)		median: 180	IQR: 150–200
Surgery blood loss (mL)		median: 100	IQR: 100–200
SLN removed			
	right	2	1–3
	left	1	1–2
Pelvic lymphadenectomy			
	right	7	5–8
	left	7	5–8
SLN detection			
	unilateral	8	19.5
	bilateral	27	65.9
	overall	35	85.4
Specimen retrieval			
	endobag	31	75.6
	trocar	10	24.4
Lymph node metastasis			
	yes	3	7.3
	no	38	92.7
FIGO stage			
	IA	21	51.3
	IB	8	19.5
	IC	1	2.4
	IIA	5	12.3
	IIB	1	2.4
	IIIB1	2	4.9
	IIIC1	3	7.3

**Table 3:** Anatomical locations.

	Right Side SLNs (N = 73)	Left Side SLNs (N = 65)
Common iliac	8 (10.9%)	3 (4.6%)
Presacral	1 (1.6%)	0 (0%)
External iliac	34 (46.5%)	30 (40.6%)
Internal iliac	10 (13.6%)	7 (10.8%)
Obturator	20 (20.7%)	23 (38.5%)

**Table 4:** Lymph node metastasis (per patient).

	Pelvic Lymphadenectomy Positive	Pelvic Lymphadenectomy Negative	Total
SLNB positive	0	3	3
SLNB negative	0	38	38
Total	0	41	41

## Data Availability

The data presented in this study are available on request from the corresponding author, due to restrictions from the Institutional Review Board.

## Abbreviations

The following abbreviations are used in this manuscript:

EC	Endometrial Cancer
OS	Overall Survival
SLNB	Sentinel Lymph Node Biopsy
SLN	Sentinel Lymph Node
ICG	Indocyanine Green
ESGO	European Society of Gynaecological Oncology
GESEA	Gynaecological Endoscopic Surgical Education and Assessment
FIGO	International Federation of Obstetrics and Gynecology
CT	Computed Tomography
D&C	Dilatation and Curettage
H&E	Hematoxylin and Eosin
ITCs	Isolated Tumor Cells
PS	Performance Status
ASA	American Society of Anesthesiologists Physical Status Classification System
NPV	Negative Predictive Value
PPV	Positive Predictive Value
FNR	False Negative Rate
BMI	Body Mass Index
